# MicroRNA‐374c‐5p inhibits the development of breast cancer through TATA‐box binding protein associated factor 7‐mediated transcriptional regulation of DEP domain containing 1

**DOI:** 10.1002/jcb.28803

**Published:** 2019-06-04

**Authors:** Shuai Hao, Wuguo Tian, Yi Chen, Lingli Wang, Yan Jiang, Bo Gao, Donglin Luo

**Affiliations:** ^1^ Department of Breast, Thyroid Surgery Daping Hospital, Army Medical University Chongqing China

**Keywords:** breast cancer, DEP domain containing 1, miR‐374c‐5p, TATA‐box binding protein‐associated factor 7

## Abstract

Breast cancer is the most pervasive cancer tormenting women, with increasing incidence and mortality rates year after year. MicroRNAs (miRNAs) with abnormal expression has various effects in biological processes and progression in diverse tumors. Nevertheless, it is vitally crucial for us to inspect more underlying molecular mechanisms for the therapy of patients with breast cancer. In the paper, we inquired the expression level and potential regulation mechanism of miR‐374c‐5p in breast cancer. Our research found out that miR‐374c‐5p was low‐level expressed in breast cancer. Upregulation of miR‐374c‐5p repressed cell proliferation, migration, and also epithelial‐mesenchymal transition (EMT), and induced cell apoptosis of breast cancer cells. Further, we concluded that miR‐374c‐5p interacted with TAF7 and downregulated its expression. Moreover, miR‐374c‐5p modulated DEP domain containing 1 (DEPDC1) through mediating TAF7. Finally, rescue assays represented that miR‐374c‐5p suppressed breast cancer development via TAF7‐mediated transcriptional regulation of DEPDC1. We uncovered that overexpressed miR‐374c‐5p inhibited the development of breast cancer via TAF7‐regulated transcriptional regulation of DEPDC1, which may be a novel and vital proportion of cancer diagnosis and treatment strategies.

## 1 INTRODUCTION

Breast cancer is one recurrent cancer and accounts for cancer‐related death torturing women in the world.^1^ It is highly heterogeneous,^2^ with increasing incidence and mortality rates.^3^ Although strategies targeting breast cancer have already been improved, systemic treatments to guard against metastasis are of less efficacy.^1^, ^4^ So it is essential to figure out even more effective therapies to control the initiation and progression of breast cancer.

MicroRNAs (miRNAs) are acknowledged as a family of small noncoding RNAs less than 30 nucleotides in length,^5^ whose dysregulation plays a pivotal role in cancers^6^ and even the regulation of biological processes in cancers, such as cell proliferation,^7^ cell cycle,^8^ differentiation,^9^ and apoptosis.^10^ miRNAs function as crucial controllers of gene expression, particularly as posttranscriptional controllers of messenger RNAs (mRNAs) via degrading mRNA or restraining translation.^11^ For example, miR‐422a targets PLP2 to repress breast carcinoma stem cells^9^; miR–483‐5p boosts the proliferative and invasive abilities in prostate cancer through downregulating RBM5^12^; miR‐378 inhibits the proliferation, invasion, and migration of colon cancer cells through decreasing SDAD1^7^; miR‐455‐5p inhibits physiological processes of ESCC cells by reducing Rab31.^13^ Since miR‐374c‐5p has merely been researched in cervical cancer, acting as a tumor suppressor in activities of cell mobility,^14^ the character of miR‐374c‐5p in breast carcinoma deserves undoubtedly careful verification.

Transcription is made up of a round of highly stated steps: the assembly of a preinitiation complex in the promoter region nucleated with TFIID for the initiation, extension, and termination.^15^ TATA‐box binding protein‐associated factor 7 (TAF7), an ingredient of the TFIID complex, binds with and furtherly controls the activities of transcription factors modulating RNA polymerase II progression.^16^

DEP domain containing 1 (DEPDC1), naming DEP domain containing 1, has been recognized to be an oncogene resulting in human cancers, containing nasopharyngeal carcinoma,^17^ hepatocellular carcinoma,^18^ prostate carcinoma,^19^ bladder carcinoma,^20^ and lung cancer.^21^ Besides, DEPDC1 is required for biological processes of tumors, involving cell proliferation,^22^ apoptosis,^21^ cycle,^17^ and metastasis.^18^ However, the role of DEPDC1 played in breast cancer was not inspected previously.

The present work inquired the expression pattern and latent mechanism of miR‐374c‐5p in breast cancer. Our findings confirmed initially miR‐374c‐5p was low‐level expressed in breast cancer and its upregulation inhibited breast cancer progression. Furthermore, miR‐374c‐5p modulated DEPDC1 through mediating TAF7. In conclusion, miR‐374c‐5p suppressed the development of breast cancer via targeting TAF7 to mediate DEPDC1, suggesting the part of miR‐374c‐5p as a latent therapeutic target for breast cancer patients.

## 2 MATERIALS AND METHOD

### 2.1 Cell culture

From the Cell Bank of the Chinese Academy of Sciences (Shanghai, China), breast cancer cell lines (BT‐549, MDA‐MB‐231, and BT474) and normal breast epithelial cell (MCF‐10A) were gained. BT‐549 cells were grown with roswell park memorial institute (RPMI) 1640 medium (Gibco, Thermo Fisher Scientific, Waltham, MA), which contains 10% fetal bovine serum (FBS, Thermo Fisher Scientific, Inc., Waltham, MA), 100 mg/mL streptomycin, and 100 U/mL penicillin. Normal MCF‐10A, MDA‐MB‐231, and BT474 cells were cultivated in Dulbecco's modified Eagle's medium (DMEM; HyClone) with 10% FBS. The cells were maintained at 37°C in a damp incubator including 5% CO_2_.

### 2.2 Cell transfection

Invitrogen (Invitrogen, Camarillo, CA) was used to synthesize miR‐374c‐5p mimic, TAF7‐pcDNA3.1, and DEPDC1‐pcDNA3.1 with NC‐mimic and pcDNA3.1 as negative controls. All vectors were gained from OBIO Company (Shanghai, China). The transfection was conducted by Lipofectamine 2000 (Invitrogen, Thermo Fisher Scientific, Inc.) following the recommendations of manufacturers.

### 2.3 Quantitative real‐time PCR

Total RNA was harvested by TRIzol reagent (Invitrogen, Camarillo, CA), followed by reverse transcription with the PrimeScript RT Reagent Kit (TaKaRa, Tokyo, Japan) based on the manufacturer's directions. Genes including miR‐374c‐5p, TAF7, and DEPDC1 were analyzed in triplicate by quantitative real‐time PCR (qRT‐PCR) experiments in an ABI PRISM 7500 sequence detection PCR System (Applied Biosystems, Foster City, CA). The fold changes of researched genes were separately normalized to glyceraldehyde 3‐phosphate dehydrogenase (GAPDH) and U6 using the $2^{-\Delta \Delta C_{t}}$ method. The primers were as follows: 5′‐ATAATACAACCTGCTAAGTGC‐3′ (forward) and 5′‐GAACATGTCTGCGTATCTC‐3′ (reverse) for miR‐374c‐5p; 5′‐GGGAAAGTTGTCTCCGGTGT‐3′ (forward) and 5′‐CATGAGGCGCATCGTCTTTG‐3′ (reverse) for TAF7; 5′‐GGTGTGCCATCCCTAGAAGA‐3′ (forward) and 5′‐CATTGCTTCTTGGCCAATTT‐3′ (reverse) for DEPDC1; 5′‐AGTGGCAAAGTGGAGATT‐3′ (forward) and 5′‐GTGGAGTCATACTGGAACA‐3′ (reverse) for GAPDH; 5′‐CTCGCTTCGGCAGCACATATACT‐3′ (forward) and 5′‐ACGCTTCACGAATTTGCGTGTC‐3′ (reverse) for U6.

### 2.4 MTT assay

To evaluate cell proliferation, cells (2 × 10^4^) were incubated in 96‐well dishes, each well of which was added with 100 µL DMEM mixed with 0.5 g/L 3‐(4,5‐dimethylthiazol‐2‐Yl)‐2,5‐diphenyltetrazolium bromide (MTT) (Thermo Fisher Scientific, Inc., Waltham, MA). Thereafter cells were cultivated understated time points of 24, 48, 72, or 96 hours. Then add dimethyl sulfoxide (50 µL; Sigma) after the removal of the medium. Postincubation for 10 minutes at 37°C, the optical density was evaluated with a plate reader (Bio Tek Instruments Inc) at 490 nm.

### 2.5 Transwell assay

Transwell migration assay was implemented using 24‐well Boyden chambers (Corning Incorporated, Corning, NY) without prepared Matrigel. After the transfection, cells were first suspended in nonserum media and afterward loaded onto the upper chambers. Chambers were placed into the wells of a 24‐well plate including media with 10% FBS at 37°C. Remained cells on the upper side were cleared through a swab. Migrated cells were fastened and stained by 0.5% crystal violet, and finally calculated from at least four random microscopic fields.

### 2.6 Flow cytometry

For cell apoptosis analysis, an Annexin‐V‐FITC/propidium iodide (PI) apoptosis detection kit (BD Biosciences, San Jose, CA) was used. Digest cells in logarithmic phase using an enzyme, centrifuging for 10 minutes at 800 rpm. Then immobilize cells overnight at −20°C and stained them through 100 μL Annexin‐V‐Fluos. Finally, at room temperature, 150 μL buffer which contains 10 μL PI and 10 μL annexin was applied to culture cells for 7 minutes in the dark and the result was examined by flow cytometry (BD FACSCalibur; BD Biosciences, San Jose, CA). The apoptosis rate is counted based on the ratio of Annexin‐V‐FITC‐positive cells to PI‐negative cells.

### 2.7 Luciferase reporter assay

The TAF7 full fragments or mutant (Mut) with the potential miR‐374c‐5p‐binding site in TAF7 were constructed and cloned into pGL3 plasmids (Promega Corporation, Fitchburg, WI) following firefly luciferase gene, named as TAF7‐wild type (WT) and TAF7‐Mut. Similarly, the promoter region of DEPDC1 including putative TAF7‐binding site was synthesized and inserted into pGL3 plasmids, given the name of pGL3‐DEPDC1‐promoter. Cells were cotransfected with luciferase reporter plasmids (400 ng), Renilla luciferase reporter vector (pRL‐TK) (50 ng) and corresponding miR‐374c‐5p mimic or NC‐mimic as well as pcDNA3.1‐TAF7 or pcDNA3.1 (50 nM) using Lipofectamine 2000. After the harvest of cells posttransfection, the luciferase activities were evaluated by the Dual‐Luciferase Reporter Assay Kit (Promega Corporation, Fitchburg, WI), normalized to Renilla luciferase activities.

### 2.8 RNA immunoprecipitation

RNA immunoprecipitation (RIP) was implemented through the EZ‐Magna RIP RNA‐binding protein immunoprecipitation kit (EMD Millipore, Billerica, MA). Cell lysis was produced by RIP lysis buffer, 100 μL of which was cultivated using RIP buffer including magnetic beads with the conjugation of anti‐Ago2 (5 μg, EMD Millipore, Billerica, MA) and anti‐ Immunoglobulin G (IgG; Millipore, Billerica, MA). Samples were grown together with proteinase K buffer and then coprecipitated RNA was separated from beads in 1 mL of TRIzol RNA extraction reagent in accordance with the handbooks of the provider.

### 2.9 Chromosome immunoprecipitation assay

BT‐549 and MDA‐MA‐231 cells were used for chromosome immunoprecipitation (ChIP) assay using the EZ ChIP Chromatin Immunoprecipitation Kit (Millipore) under the provider's guidelines. Postsonicating cross‐linked chromatins into the debris of 200 to 1000 bp, anti‐TAF7 antibodies (Abcam) were utilized to immunoprecipitate protein‐DNA complexes. Anti‐immunoglobulin G (IgG; Millipore) served as a negative control antibody. The fragment of DEPDC1 promoter containing the TAF7 sites was amplified from the precipitated DNA samples. qRT‐PCR was conducted to assess the amount of immunoprecipitated DNA.

### 2.10 Western blot

3 × 10^5^ cells were placed into six‐well culture dishes to achieve 70% to 80% confluency, washed by phosphate‐buffered saline and suspended in RIPA buffer (100 μL; Pierce, Dallas, TX). The concentration of supernatant protein was determined with the BCA protein assay kit (Pierce). The supernatant samples involving 30 μg total protein were dissolved by 10% or 12.5% sodium dodecyl sulfate‐polyacrylamide gel electrophoresis, then transferred onto Immobilon‐P polyvinylidene difluoride membranes (Millipore) via electroblotting, subsequently probed with primary antibodies. Membranes were cultivated with horseradish peroxidase‐conjugated secondary antibodies. The primary antibodies were anti‐E‐cadherin (Santa Cruz Biotechnology), anti‐N‐cadherin (Santa Cruz Biotechnology, Santa Cruz, CA), anti‐vimentin (GeneTex, Irvine, CA), anti‐DEPDC1 (Abcam), and anti‐GAPDH (Cell Signaling Technology, Danvers, MA) antibodies.

### 2.11 Statistical analysis

Dissections of statistics were analyzed using the SPSS version 19.0 (SPSS Inc., Chicago, IL), expressed as mean ± SD and thought as significant when *P* < 0.05. Comparisons were calculated by the Student *t* test or analysis of variance according to a number of groups. Every experiment was repeated three times.

## 3 RESULTS

### 3.1 miR‐374c‐5p was low‐expressed in breast cancer, whose overexpression inhibited breast cancer development

**Figure 1:**
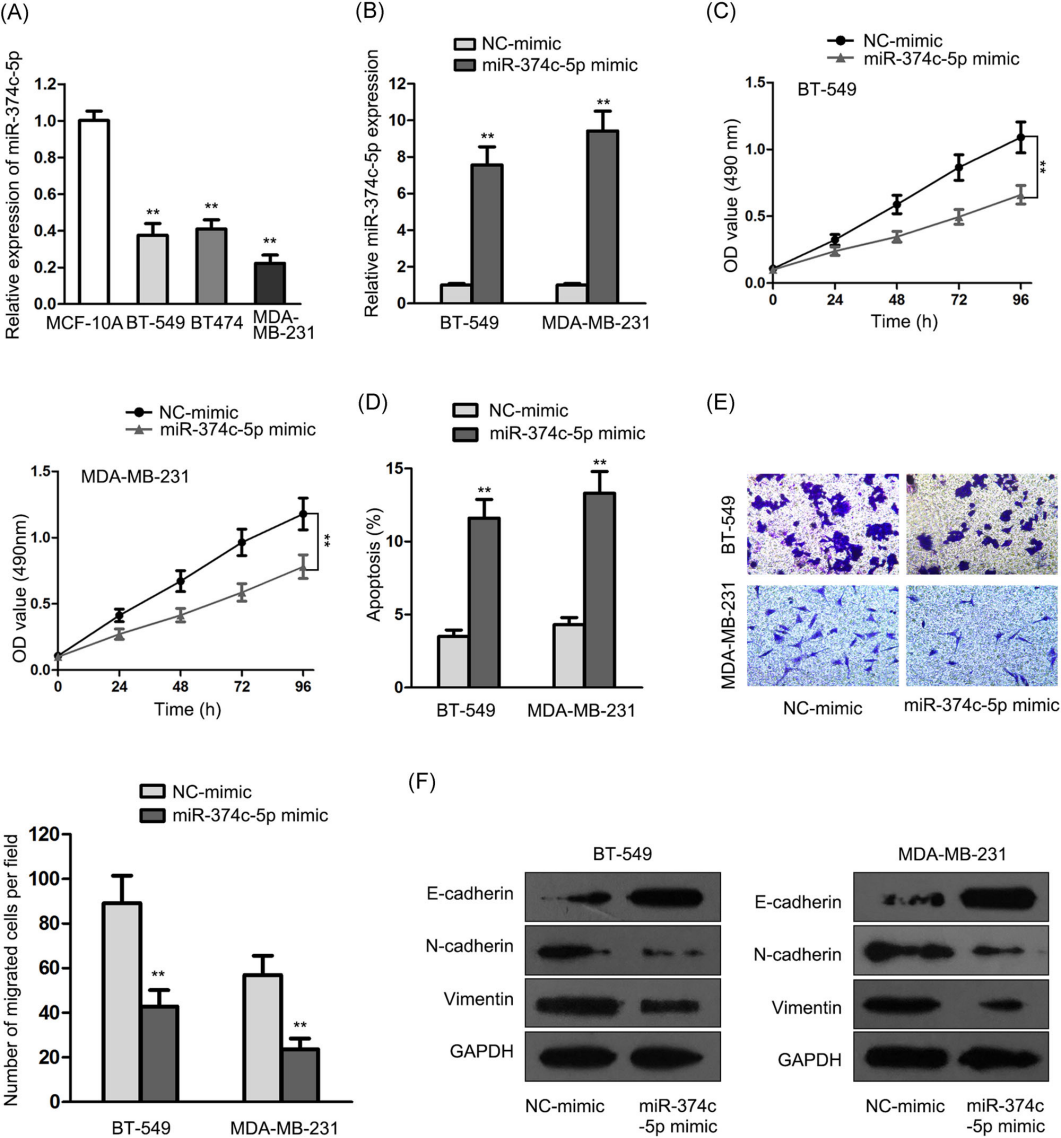
**:** miR‐374c‐5p was expressed at low levels and its overexpression inhibited breast cancer progression. A, qRT‐PCR analysis of miR‐374c‐5p expression in breast cancer cell lines (BT‐549, BT474, and MDA‐MB‐231) and normal breast epithelial cell line MCF‐10A. B, miR‐374c‐5p mimic was used to overexpress miR‐374c‐5p in both BT‐549 and MDA‐MB‐231 cells. The expression level of miR‐374c‐5p was significantly high as estimated by qRT‐PCR assay. C, MTT assay was performed to test cell proliferation of BT‐549 and MDA‐MB‐231 cells. D, Cell apoptosis rate of BT‐549 and MDA‐MB‐231 cells was measured by flow cytometry. E, Transwell analysis of cell migration in BT‐549 and MDA‐MB‐231 cells. F, Western blot was used to detect the levels of EMT‐associated proteins (E‐cadherin, N‐cadherin, and vimentin). qRT‐PCR, quantitative real‐time PCR

For the investigation of the feasible participation of miR‐374c‐5p in breast cancer initiation and progression, we made an analysis of miR‐374c‐5p expression in breast cancer cells (BT‐549, BT474, and MDA‐MB‐231) and normal breast epithelial cells (MCF‐10A) using qRT‐PCR assay. the qRT‐PCR result showed miR‐374c‐5p was lowly expressed in breast cancer cell lines (Figure 1A). We performed gain‐of‐function assays to research the character of miR‐374c‐5p in BT‐549 cells and MDA‐MB‐231 cells which exhibited lower miR‐374c‐5p expression among the cultured breast cancer cell lines. miR‐374c‐5p levels were markedly overexpressed in miR‐374c‐5p mimic transfected BT‐549 and MDA‐MB‐231 cells as detected by qRT‐PCR (Figure 1B). In MTT assay, cell proliferation of miR‐374c‐5p mimic stably transfected cells was dramatically inhibited in comparison with the control group (Figure 1C). In flow cytometry analysis, cell apoptosis rates of BT‐549 and MDA‐MB‐231 cells infected with miR‐374c‐5p mimic were obviously more than that of the control group (Figure 1D). Besides, cells with miR‐374c‐5p overexpression displayed clear repression in cell migration as detected by transwell assay (Figure 1E). Furthermore, E‐cadherin levels were augmented while N‐cadherin and vimentin levels were lessened when miR‐374c‐5p was overexpressed, as tested by Western blot (Figure 1F). All data indicated that in breast cancer, the overexpression of miR‐374c‐5p restrained cell proliferation, induced cell apoptosis, hindered migration, and EMT process.

### 3.2 miR‐374c‐5p interacted with TAF7 and further downregulated its expression

**Figure 2:**
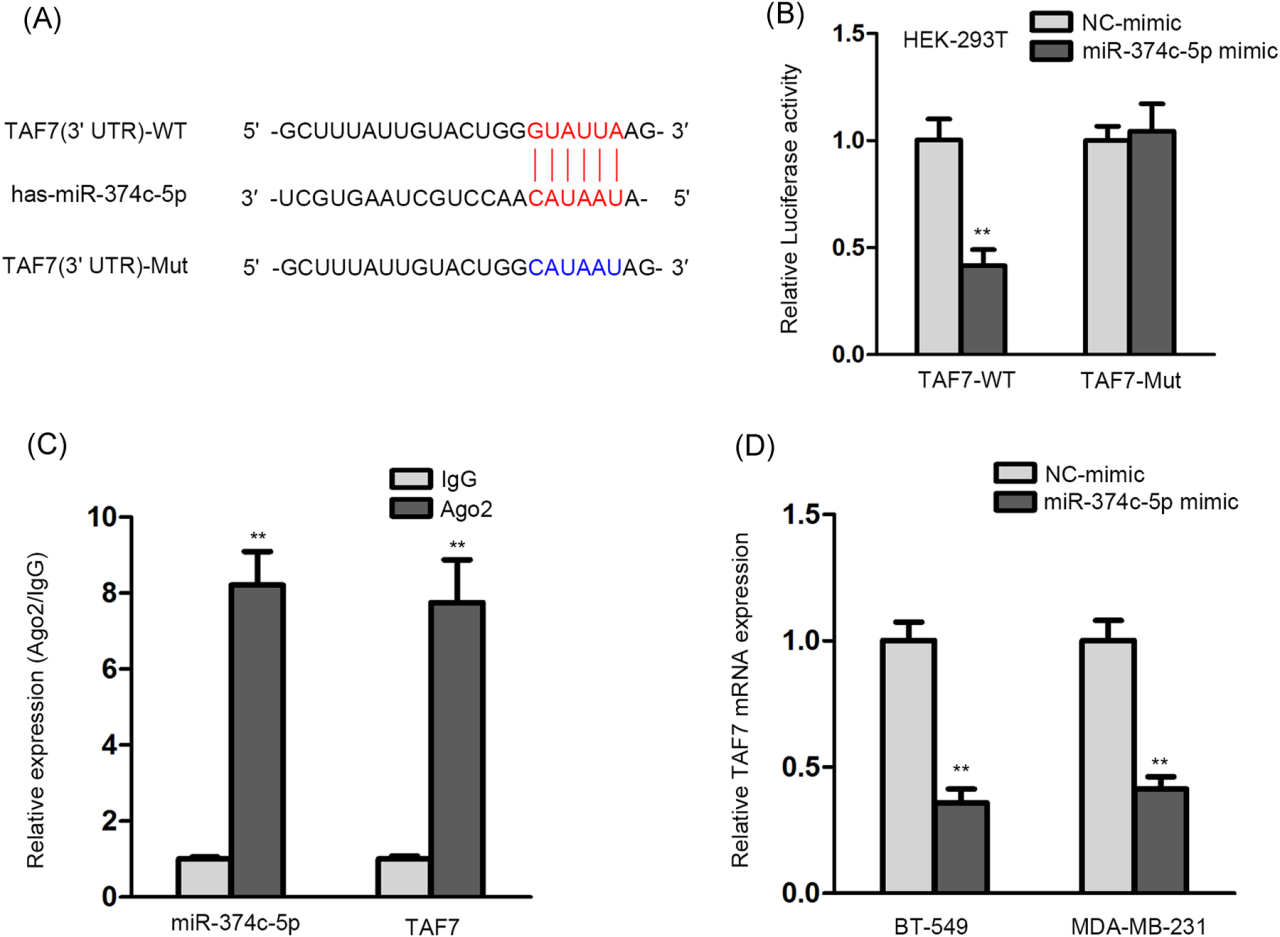
**:** miR‐374c‐5p interacted with TAF7 and further downregulated its expression. A, The putative binding sites between miR‐374c‐5p and TAF7 from TargetScan website. B, Luciferase reporter assay was conducted to confirm the combination of miR‐374c‐5p and WT TAF7 in HEK‐293T cell. C, RIP assay was utilized to affirm the binding between miR‐374c‐5p and TAF7. D, The impact of miR‐374c‐5p overexpression on the mRNA level of TAF7 was examined by qRT‐PCR experiment in BT‐549 and MDA‐MB‐231 cells. mRNA, messenger RNA; qRT‐PCR, quantitative real‐time PCR; TAF7, TATA‐box binding protein‐associated factor 7

It is generally acknowledged that miRNAs exert their function via modulating target genes.^23^, ^24^ From TargetScan, we found TAF7, with a potential binding site with miR‐374c‐5p (Figure 2A). As described in Figure 2B, the luciferase activity of WT TAF7 was lessened in the miR‐374c‐5p mimic group but had no obvious variation in the NC group. Moreover, in RIP assay, the complex precipitated by anti‐Ago2 exhibited relatively high expression of miR‐374c‐5p and TAF7 (Figure 2C), further affirming the interaction between miR‐374c‐5p and TAF7. Furthermore, qRT‐PCR experiment elucidated that TAF7 mRNA expression was knocked down under miR‐374c‐5p upregulation (Figure 2D). Briefly, miR‐374c‐5p combined with TAF7 and regulated its expression negatively.

### 3.3 miR‐374c‐5p modulated DEPDC1 by mediating TAF7

**Figure 3:**
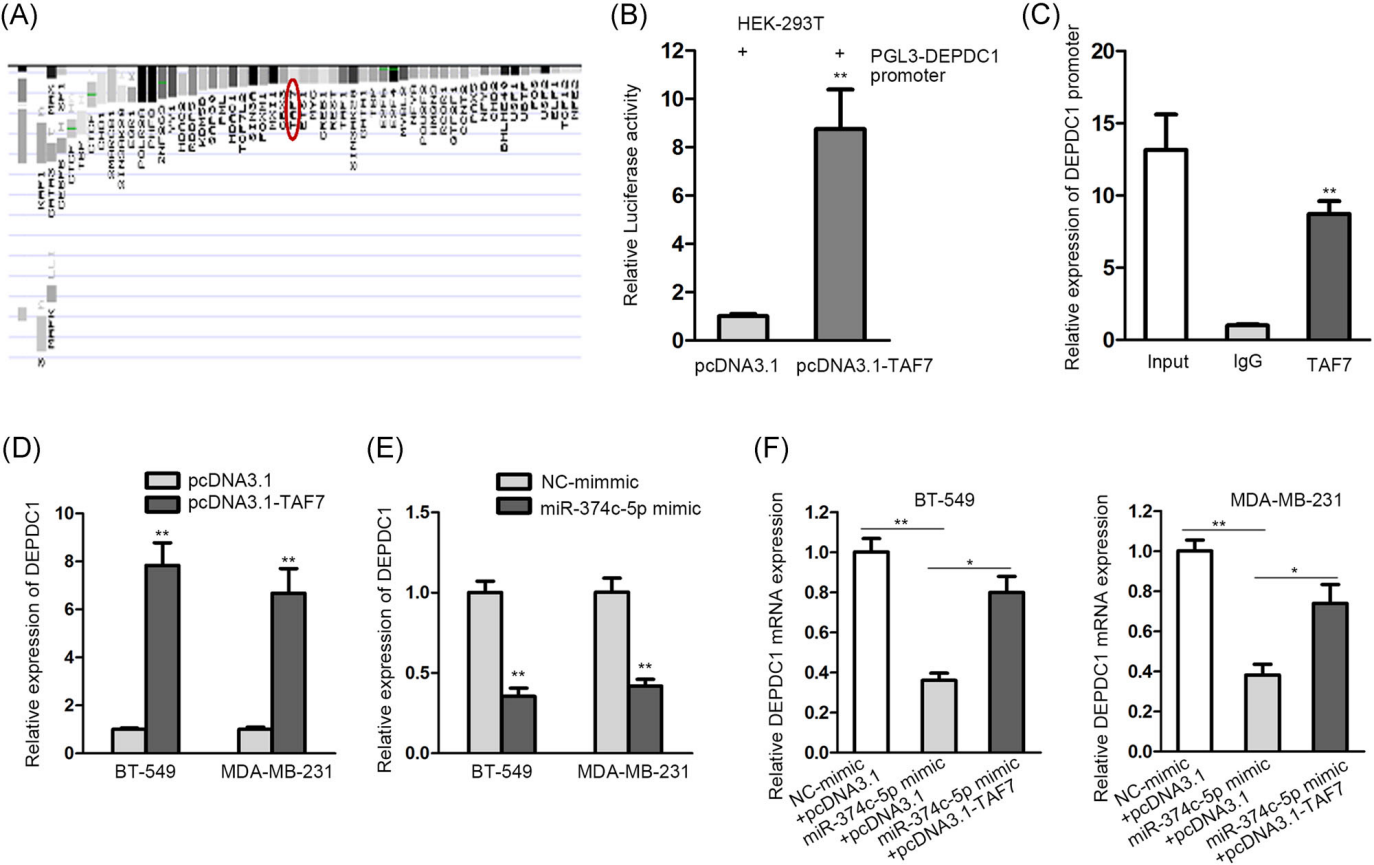
**:** miR‐374c‐5p modulated DEPDC1 by mediating TAF7. A, The potential transcriptional regulation of TAF7 on DEPDC1 were gained from the UCSC Genome Browser. B, In HEK‐293T cell, the binding between TAF7 and DEPDC1 promoter was verified by luciferase reporter assay. C, ChIP results of the combination of TAF7 and DEPDC1 promoter. D, qRT‐PCR detection of DEPDC1 expression in BT‐549 and MDA‐MB‐231 cells under pcDNA3.1‐TAF7 treatment. E, DEPDC1 expression was detected by qRT‐PCR assay after miR‐374c‐5p was overexpressed in BT‐549 and MDA‐MB‐231 cells. F, The qRT‐PCR result of DEPDC1 mRNA expression in BT‐549 and MDA‐MB‐231 cells after cotransfection of miR‐374c‐5p mimic and pcDNA3.1 or miR‐374c‐5p mimic and pcDNA3.1‐TAF7, in comparison to the control group. DEPDC1, DEP domain containing 1; qRT‐PCR, quantitative real‐time PCR; TAF7, TATA‐box binding protein‐associated factor 7

Further on, we probed into the underlying mechanism of miR‐374c‐5p and TAF7. As illustrated before, miR‐374c‐5p can regulate gene expression in tumors and TAF7 can transcriptionally modulate gene expression. Hence, we supposed that miR‐374c‐5p might mediate TAF7 to regulate gene expression. DEPDC1 was selected as it was claimed to play an oncogenic function in carcinomas^19^, ^22^, ^25^ and affect biological activities, such as cell proliferation,^22^ apoptosis,^21^ and metastasis.^18^ As exhibited in Figure 3A, we obtained the predicted transcriptional regulation of TAF7 on DEPDC1 from UCSC Genome Browser. The result of luciferase reporter assay validated the upregulation of TAF7 purely enhanced the luciferase activity of WT DEPDC1 promoter (Figure 3B). In the ChIP assay, the promoter region of DEPDC1 was merely enriched in the compound targeted by anti‐TAF7 (Abcam, Cambridge, MA), antibody (Figure 3C). When TAF7 was overexpressed, DEPDC1 mRNA expression was also clearly increased (Figure 3D). Unsurprisingly, DEPDC1 mRNA expression was repressed after miR‐374c‐5p was upregulated (Figure 3E). To prove that miR‐374c‐5p regulated DEPDC1 by modulating TAF7, cells were cotreated with miR‐374c‐5p mimic + pcDNA3.1 or miR‐374c‐5p mimic + pcDNA3.1‐TAF7, followed by qRT‐PCR detection. The mRNA level of DEPDC1 was reduced under miR‐374c‐5p overexpression, which could be alleviated under TAF7 upregulation. Taken together, miR‐374c‐5p modulated DEPDC1 through mediating TAF7 (Figure 3F).

### 3.4 miR‐374c‐5p influenced the tumorigenesis and progression of breast cancer through regulating DEPDC1

**Figure 4:**
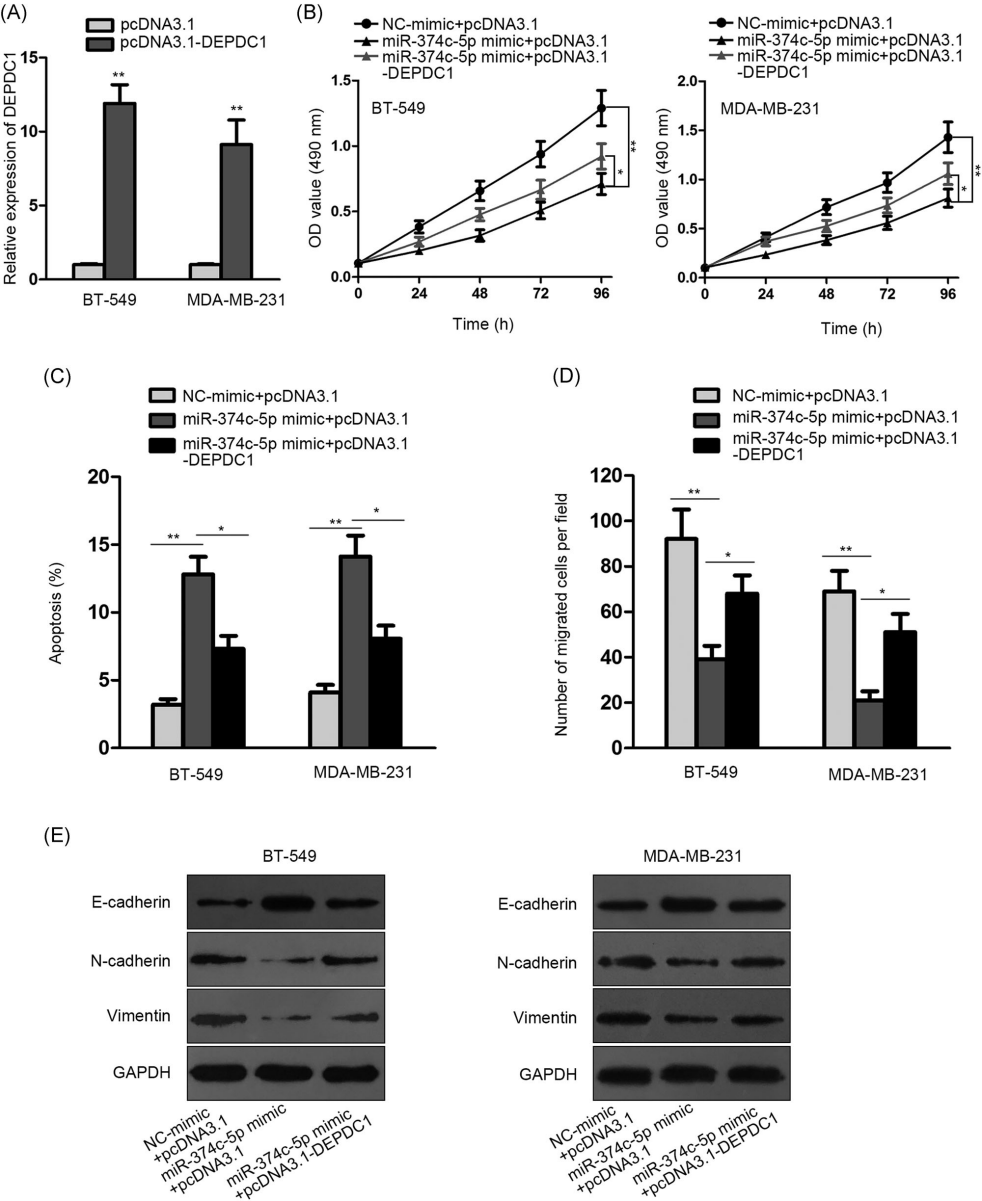
**:** miR‐374c‐5p influenced the tumorigenesis and progression of breast cancer through regulating DEPDC1. miR‐374c‐5p mimic and pcDNA3.1 or miR‐374c‐5p mimic and pcDNA3.1‐DEPDC1 were cotransfected into BT‐549 and MDA‐MB‐231 cells. A, DEPDC1 expression in BT‐549 and MDA‐MB‐231 cells with pcDNA3.1‐DEPDC1 transfection was tested via qRT‐PCR assay. B, MTT analysis of the proliferative capacity of BT‐549 and MDA‐MB‐231 cells. C, Flow cytometry was implemented to evaluate the apoptosis rate of BT‐549 and MDA‐MB‐231 cells. D, Cell migration of BT‐549 and MDA‐MB‐231 cells were estimated by transwell experiment. E, Western blotting detected the EMT‐associated proteins levels in BT‐549 and MDA‐MB‐231 cells. DEPDC1, DEP domain containing 1; qRT‐PCR, quantitative real‐time PCR

To ensure the mechanism underlying miR‐374c‐5p in breast cancer, rescue experiments were performed. Tumor cells were cotreated with miR‐374c‐5p mimic + pcDNA3.1 or miR‐374c‐5p mimic + pcDNA3.1‐DEPDC1, along with NC‐mimic + pcDNA3.1 (NC) (Figure 4A). MTT assay disclosed that miR‐374c‐5p overexpression inhibited cell proliferation while the high expression of DEPDC1 alleviated this impact of miR‐374c‐5p on cell proliferation (Figure 4B). According to flow cytometry evaluation, cell apoptosis was restrained under the upregulation of miR‐374c‐5p, which could be partially abrogated by DEPDC1 overexpression (Figure 4C). In transwell assay, high‐expressed DEPDC1 neutralized the repression of miR‐374c‐5p on cell migration (Figure 4D). Finally, Western blotting demonstrated that the protein level of E‐cadherin was raised under miR‐374c‐5p upregulation but reversed in part after the addition of DEPDC1, which is contrary to that of N‐cadherin or vimentin (Figure 4E). To sum up, miR‐374c‐5p affected the tumorigenesis and progression of breast cancer via regulating DEPDC1.

## 4 DISCUSSION

Breast cancer is the primary reason of cancer‐related mortality happening among women globally, with increasing incidence and mortality rates.^3^ Despite numerous advances in breast cancer treatment have been produced, therapies available were still needed for limited efficacy.^3^, ^4^ Hence, it is vital for us to figure out more effective therapies preventing against breast cancer. The initiation and progression of breast cancer are related to various factors, hinting that molecular‐targeted therapy can offer a specific understanding of the pathogenesis of breast cancer.

Prevenient studies have declared that miRNAs are important modulators of gene expression at the posttranscriptional level via mRNAs degradation or translation inhibition.^11^ The dysregulation of miRNAs plays an important part in the modulation of biological and physiological processes of different carcinomas.^26^, ^27^

miR‐374c‐5p plays a tumor‐suppressive function in cervical cancer carcinogenesis and progression, affecting the migratory and invasive properties of cervical cancer.^14^ Herein, the function of miR‐374c‐5p was firstly explored in breast cancer. In our study, low‐expressed miR‐374c‐5p was disclosed in breast cancer. Moreover, miR‐374c‐5p overexpression inhibited cell proliferation, promoted cell apoptosis, and impeded migration and EMT of breast cancer cells.

As miRNAs have been distinguished to play vitally crucial parts in carcinomas through modulating gene expression,^11^ we further sought for the downstream genes. Fortunately, we gained the binding site between miR‐374c‐5p and TAF7 from TargetScan. TAF7, a part of the TFIID complex, combines with and mediates the activities of transcription factors which adjust RNA polymerase II progression.^16^ Our research uncovered that miR‐374c‐5p combined with TAF7 and regulated its expression in a negative way.

Furthermore, we obtained DEPDC1 from UCSC Genome Browser, which is under the transcriptional regulation function of TAF1. Intriguingly, DEPDC1 gives play to cancerogenic function in human tumors, containing nasopharyngeal carcinoma,^17^ hepatocellular carcinoma,^18^ prostate carcinoma,^22^ bladder carcinoma,^20^ and lung cancer.^21^ Previous studies have elaborated that DEPDC1 can also affect biological processes of tumors, such as cell proliferation,^22^ apoptosis,^19^ cycle,^17^ and metastasis.^18^ Consistently, the present investigation confirmed that miR‐374c‐5p regulated DEPDC1 through mediating TAF7, verifying the hidden mechanism of miR‐374c‐5p/TAF7/DEPDC1 axis in breast cancer.

Finally, the mechanism underlying miR‐374c‐5p was verified by performing rescue assays. miR‐374c‐5p modulating TAF7‐regulated DEPDC1 indeed influenced the carcinogenesis and progression of breast cancer.

Here, we drew a conclusion that miR‐374c‐5p inhibited breast cancer development via TAF7‐mediated transcriptional regulation of DEPDC1. The current paper implied the promising biomarker meaning of miR‐374c‐5p in breast cancer.

## CONFLICT OF INTERESTS

No latent conflict of interest was disclosed.

## AUTHOR CONTRIBUTIONS

SH was in charge of designing and carrying out this study. WT, YC, and LW were responsible for the collection of experimental materials and the records of experimental results. YJ, BG, and DL, took charge of statistical analysis and article writing. Each author conduced to this study and provided valuable advice for this manuscript.
